# Copper Poisoning, a Deadly Hazard for Sheep

**DOI:** 10.3390/ani12182388

**Published:** 2022-09-13

**Authors:** Marta Borobia, Sergio Villanueva-Saz, Marta Ruiz de Arcaute, Antonio Fernández, María Teresa Verde, José María González, Teresa Navarro, Alfredo A. Benito, José Luis Arnal, Marcelo De las Heras, Aurora Ortín

**Affiliations:** 1 Animal Pathology Department, Instituto Agroalimentario de Aragón-IA2, Facultad de Veterinaria, Universidad de Zaragoza-CITA, C/Miguel Servet 177, 50013 Zaragoza, Spain; 2 EXOPOL S.L., Pol. Río Gállego D/S, San Mateo de Gállego, 50840 Zaragoza, Spain

**Keywords:** chronic copper poisoning, acute copper poisoning, copper toxicity, hepatic copper accumulation, sheep, lamb, anaemia

## Abstract

**Simple Summary:**

Sheep are very susceptible to copper intoxication, a deadly disease that causes significant economic losses worldwide. Two types of copper poisoning can occur depending on the chronic or acute exposure to copper. Chronic toxicosis is the most common form and is developed after a long subclinical period of copper accumulation in the liver. When the capacity of the liver for copper storage is exceeded, a sudden release of copper into the blood causes severe haemolysis and the death of the animals. Acute copper poisoning is much less frequent and appears following the accidental administration or ingestion of toxic amounts of copper. Collapse and death occur shortly after parenteral administration, whereas acute oral exposure to copper causes severe gastroenteritis followed by shock and death. In this review, we summarise the available information on the aetiology, epidemiology, pathogenesis, clinical features, diagnosis, treatment and prevention of sheep copper poisoning.

**Abstract:**

Copper (Cu) is an essential microelement for animals. However, sheep are particularly susceptible to Cu intoxication, a deadly disease reported worldwide. The risk of developing this poisoning is higher in vulnerable breeds and in intensively managed lambs or milk sheep. Two types of Cu intoxication can occur depending on the chronic or acute exposure to Cu. In chronic Cu poisoning (CCP), the most common form, Cu is accumulated in the liver during a subclinical period. A low intake of Cu antagonists (molybdenum, sulphur, iron, or zinc) favours Cu accumulation. The sudden release of Cu into the blood causes acute haemolysis with anaemia, haemoglobinuria, jaundice and death within 1–2 days. Acute Cu poisoning is related to the accidental administration or ingestion of toxic amounts of Cu. Acute oral exposure to Cu causes severe gastroenteritis, shock and death. Collapse and death occur shortly after parenteral administration. The diagnosis is based on history, clinical, gross pathological, histological and toxicological findings. Treatment of sheep with severe clinical signs often has poor success but is very effective during the Cu accumulation phase. Different therapies, based on either chelating agents or Cu antagonists, have been used to treat and prevent CCP.

## 1. Introduction

Copper (Cu) has been shown to be an essential microelement for animals and plays an important role in many biological processes. It is an essential component of several important enzymes involved in the formation of connective tissue, iron metabolism, cellular respiration, protection against oxidant stress, catecholamine biosynthetic pathway, formation of myelin, melanin pigment and keratin, and it is also a key trace mineral required for effective immune response [1,2]. Therefore, a low Cu status contributes to the development of a wide range of hepatic, neurological and other types of disorders, and dietary Cu additions are routinely used in livestock production [1,2,3]. Nevertheless, Cu in excess has been shown to cause toxicity in both humans and animals [2,4,5]. Monogastrics seem to tolerate excess Cu much better than ruminants, and sheep are particularly susceptible to the effects of Cu toxicity because their Cu excretory mechanism is less efficient [1,6,7,8,9]. Because of this, Cu intoxication, also known as Cu poisoning or Cu toxicosis, is widespread in sheep flocks, mainly due to chronic exposure to Cu [10,11]. The disease has been reported in many sheep-rearing areas of the world and can cause significant economic losses due to the high mortality rates in clinically affected animals [5,10,11]. Although many factors can predispose sheep to Cu poisoning, there is evidence that the incidence of this disease is increasing as more intensive methods of sheep production are adopted, or susceptible breeds are used [11,12]. In this sense, in some countries the problem is particularly important and is considered one of the most important illnesses affecting the sheep industry [6,7,13].

In this article, we have reviewed the literature on Cu poisoning in sheep addressing the most relevant aspects of aetiology, epidemiology, pathogenesis, clinical features, diagnosis, treatment and prevention of this disease which allow a better understanding and more effective control of this deadly hazard for sheep. A bibliographic search was carried out in electronic databases including Pubmed, Web of Science and Google Scholar using the following keywords: “copper toxicosis”, “copper poisoning”, “copper intoxication”, “acute”, “chronic”, “cumulative”, “hepatic copper accumulation”, “sheep”, “lamb”, “diagnosis“, “treatment”, “prevention” and combinations of them. Other inclusion criteria were the language (English) and date of publication (between 1 January 1980, and 30 June 2022). In addition, the bibliography cited in the publications obtained from this initial search was also reviewed for additional relevant references.

## 2. Aetiology

Two types of Cu toxicosis can occur in animals: acute and chronic. Acute Cu poisoning is considered to be relatively infrequent [8,11] and is associated with overexposure to the element due to the parenteral or oral administration of excessive therapeutic Cu doses or the ingestion of toxic amounts of Cu from contaminated food or other sources of Cu [10,14,15]. In contrast, chronic Cu poisoning (CCP) is the most common form of Cu toxicosis. It is developed as the result of long-term exposure (from weeks to months) to doses above the dietary requirement or non-toxic Cu doses which accumulate in the liver [8,14,16,17], especially in those species presenting a high susceptibility [8]. This is the case of sheep, which are very vulnerable to CCP due to their inability to increase the excretion of Cu in bile in response to a higher influx of Cu into the liver [7,8,18,19,20].

It should be noted that excessive exposure to Cu is the cause of primary CCP, but secondary CCP may be associated with many factors that alter Cu metabolism by enhancing the absorption or retention of Cu. That is the case of the ingestion of certain plants (such as *Heliotropium europaeum* or *Senecia* sp.) containing toxic alkaloids, which cause metabolic liver disorders and consequently increase the liver affinity for Cu storage [12,16,21]. This type of toxicosis has been seen only in sheep and requires the ingestion of not only hepatotoxic plants but also a source of Cu [16]. The ingestion of pastures with relatively low amounts of Cu but rich in sulphates and/or poor in molybdenum (Mo) can also cause CCP due to the impact of sulphates on the absorption and storage of Mo by the plant, and the antagonistic relationship between Cu and Mo [16]. Furthermore, the plant species, the state of the soil and the application of fertilizers influence the levels of Cu, sulphur (S) and Mo in pasture and forage [6]. Interactions in the gut of ruminants between Cu and other elements from the diet such as Mo, S, zinc (Zn) or iron (Fe) affect the allowance of Cu ingested. A low intake of these Cu antagonists can also contribute to the development of CCP [7,8,10,12,22,23].

## 3. Epidemiology

Ruminants, especially sheep and, to a lesser extent, cattle, are known to be especially susceptible to Cu toxicosis. However, other species, such as pigs, poultry and horses, are more resistant [9,10,24,25]. The susceptibility to Cu varies among sheep breeds [19,26,27], which seems to be associated with the ability to excrete Cu in the bile [28]. North Ronaldsay is probably the most susceptible breed, and the Merino sheep is the most tolerant [19,26]. Cambridge breed is considered Cu-tolerant [19], whereas it has been reported that the Suffolk, Texel, Bluefaced Leicester and Charollais breeds have a higher risk of Cu poisoning [5,11,29,30,31]. Furthermore, the CCP case described by Garcia-Fernandez et al. [17] in a Segureña sheep flock suggests that this breed could also have a greater susceptibility. Genetic factors influence the response to Cu, and the gene products or proteins determine the nature of the pathological response at the cellular level. In fact, it has been demonstrated that the higher susceptibility of the mitochondria to Cu-induced oxidative stress observed in the North Ronaldsay breed compared to the Cambridge breed is due to the upregulation of mitochondrial thioredoxin-dependent peroxidase reductase (antioxidant protein-1) in the hepatic cytosol [19].

Regarding age, lambs are more susceptible to Cu toxicosis, which may be attributable to their Cu absorption capacity being higher than in adult sheep, and their biliary excretion being immature [18,32]. Digestive processes in the rumen reduce Cu absorption, and the high absorption efficiency in milk-fed lambs (they can absorb 70–80% of the Cu ingested) makes them more susceptible to Cu poisoning than weaned lambs and adults [12].

Cu intoxication is a global problem and concerns several sheep-rearing countries, such as Scandinavian countries, Canada, Australia, New Zealand, United Kingdom, USA and South Africa [6,11,31,33]. In addition, several outbreaks and cases have been described in other countries such as Spain [17], Brazil [15,31,34], Greece [35,36], Iran [37] and Turkey [38,39]. With respect to the production system, CCP is more frequent in intensive indoor conditions, housed lambs or milk sheep fed with large amounts of concentrates [12,30,35], and rarely occurs in grazing sheep under natural conditions, except in susceptible breeds or when pastures are contaminated with Cu or have very low levels of Mo [12]. Although many factors can predispose sheep to Cu poisoning, and the quantification of cases worldwide is not known by the authors, there is evidence that the incidence of this disease is increasing as more intensive methods of sheep production are adopted, or susceptible breeds are used [11,12]. The mortality rate in clinically affected animals is around 100% in acute and chronic poisoning [5,10], whereas morbidity, although it varies according to breed susceptibility, is generally significantly much lower [15,17,31,35,38]. Cu intoxication is associated with significant economic losses due to mortality, the reduction in production and/or the implementation of treatments and control measures; nevertheless, these are difficult to estimate due to the influence of several factors, such as the flock management, breed susceptibility or the production system [5,10,11,31,40,41].

The most common sources of Cu, which can lead to intoxication, are compound feeds and mineral supplements [5,8]. Feeds formulated explicitly for sheep present less risk, although they may not have sufficiently low Cu levels, especially for animals kept under intensive conditions [5]. In addition, errors in the formulation of rations or in the mixing of feed and the administration of feed formulated for Cu-tolerant species (especially swine and poultry feed being supplemented with Cu) can cause Cu poisoning in sheep [5,8,10,31,35,42]. Other sources of Cu include Cu-contaminated feedstuff, such as forage sprayed with fumigants or fungicides [10,16,38], pasture fertilised with swine manure slurry and poultry litter, which may also be ingested accidentally [5,10,35,43], or vegetation and soil contaminated as a result of industrial and mining activities [10,17,35,37,44]. The ingestion of Cu sulphate footbaths, water contaminated with fungicides, algaecides, molluscicides or containing Cu dissolved from pipping, and the use of ruminal boluses with Cu wires which can release Cu slowly, can be other sources of oral exposure to the element [5,8,15,16,38]. Furthermore, acute poisoning is very often associated with oral and parenteral overdoses of Cu salts present in preparations used for the prevention and treatment of Cu deficiency or in anthelmintic drenches [5,10,11,45]. Soluble formulations, such as Cu edetate and diethylamine oxyquinoline sulphonate (DOS), whose Cu is rapidly available, may be mortal for sheep when administered parenterally, even at recommended doses [10,46,47].

Cu toxicity depends not only on the amount of Cu ingested but also on other factors, such as the amount of Cu antagonists contained in the diet. A low intake of Cu antagonists, such as Mo, S, Fe and Zn, can contribute to the development of CCP [8,10,12,22,23]. Cu intoxication can be extremely likely if Cu and Mo are not balanced in the diet. According to the literature, with normal Cu concentrations, the risk of Cu accumulation increases as the Cu to Mo ratio in the diet increases above 6:1, CCP can occur when ratios exceed 10:1 and diets whose ratio is above 20:1 are very dangerous for sheep [8,17,43]. The imbalance of Cu, Mo and S in cereal-rich diets, which are potentially low in Mo and S, may be an important factor in the susceptibility of housed sheep to CCP [6]. The shortage of Mo in pastures containing Cu also involves a high risk of intoxication [43].

Furthermore, the bioavailability of Cu is greater in processed feedstuffs that are low in fibre than in fresh plant material [10,48,49]. The lack of access to green forage in indoor conditions can also favour chronic poisoning [17].

It has been reported that monensin interferes positively with the hepatic accumulation of Cu, and the supplementation of this additive may predispose sheep to Cu poisoning [50,51].

## 4. Pathogenesis

### 4.1. Copper Physiology in the Digestive Tract and Splanchnic Tissues

Cu requirement estimation in sheep is influenced by the age, the physiological state, and the amount of Cu antagonists in the diet (Mo, S, Fe and Zn), varying from 4.3 to 28.4 ppm on a dry matter basis (DM) [52]. Cu toxicity depends not only on the dose but also on the source and the route of administration [52], as well as the availability of Mo, S, Fe and Zn, which restrict the intestinal absorption of Cu and its storage in the liver and kidneys [52,53]. Ingested Cu accesses the digestive tract and reaches the rumen, where this element can be toxic to ruminal bacteria [54]. In addition, Mo reacts with S in the rumen leading to the formation of thiomolybdate compounds, which bind Cu, preventing its absorption [55]. Dietary Cu is absorbed once it has been reduced at the intestinal brush border into Cu^+^, which is its most reactive state [9]. Intestinal absorption mainly happens through the specific transporter Ctr1, whereas the rest is taken up by the non-specific transporter divalent metal transporter 1 (DMT1), where competition with dietary elements such as Zn and Fe may be especially significant [9,41,56]. Cu is transported inside the enterocyte through Cu chaperone proteins to be supplied to cellular requirements, and excess Cu is bound to the protein called metallothionein (MT) by the Golgi and stored inside the lysosome [9]. Upon reaching the MT-carrying capacity in the lysosome, surplus Cu from the Golgi is transported using the ATP7A secretory pathway and effluxed from the enterocyte into circulation [9]. Cu is a weak MT inducer. By contrast, MT synthesis is highly regulated by Zn, and once Zn has induced MT synthesis, Cu can efficiently compete with Zn for MT-binding sites [24,28]. The effective induction of MT by Zn in the intestine limits intestinal Cu absorption [7]. Following efflux from the enterocytes, Cu is transported to the liver bound to albumin and transcuprein, a specific Cu carrier in blood plasma [8,9]. Once Cu accesses the liver, it is incorporated into newly synthesised caeruloplasmin, the predominant Cu transporter in the systemic blood and responsible for the distribution of Cu to the tissues [9,57]. The whole molecule is absorbed when caeruloplasmin-bound Cu returns from the tissues to the liver. Caeruloplasmin is destroyed and excreted through the biliary route, and the excess hepatic Cu is exported into the bile [9]. Cells do not harbour free Cu ions in order to prevent intracellular damage. Therefore, it is necessary for Cu to remain associated with other molecules. MT binds free Cu and acts as a storage buffer protecting the cell [9,58,59]. When hepatocytes are exposed to increasing Cu concentrations, the chaperone ATP7B (from the hepatocyte secretory pathway) imports Cu in the non-toxic MT-bound form into the lysosomal lumen for temporary storage, and the exocytosis of the lysosome is induced, releasing the excess Cu into the biliary canal [9].

The critical role of MT is to contribute safely to the intracellular storage of Cu [60]. The increase in MT expression, which is regulated by metal transcription factor MTF1, enables a response to Cu influx by Cu sequestering in the lysosome; nevertheless, this ability is higher in monogastrics than in ruminants, which are especially susceptible to Cu toxicosis [4,9,24,61]. It has been described that the hepatic MT levels are about 200 mg/kg in sheep and cattle, whereas these concentrations are more elevated in pigs and dogs (500–600 mg/kg) [24]. The ovine species has a limited capacity to increase the synthesis of MT in response to ascending Cu concentration, to accumulate MT-bound Cu in the liver and to increase the excretion of Cu in bile in response to increased intake [3,7,8,9,18,19,28,61]. In the case of a large influx of Cu into the liver, the capacity of the MT to bind Cu and the ability of the lysosome to sequester MT-bound Cu may be exceeded, and consequently, the unbound element is accumulated mainly in the nucleus and in the cytosol, causing cell damage [3,9]. Thus, sheep are sensitive to Cu oversupply due to their inability to adapt to Cu influx [9].

### 4.2. Chronic Copper Poisoning

CCP can be developed when sheep consume 3.5 mg of Cu per kg of body weight daily for a long period. The maximum concentration tolerated in feed is 25 ppm [10]. However, as stated before, different factors depending on the animal and the environment predispose to Cu accumulation in the liver and the consequent liver damage. When damage is severe, hepatic necrosis develops, and Cu is released into the bloodstream. This Cu mobilization is triggered by a stressful event, any stressful condition such as handling, shearing, transportation, exertion, a drop in the diet ration, lactation, weather changes or concurrent diseases, predispose to the release of the hepatic Cu [8,10,11,16,36,43]. Free Cu ions oxidise haemoglobin to methaemoglobin and cause oxidative damage to the red blood cells membrane, and rapid haemolysis [5,8] due to the formation of Heinz bodies, aggregates of denatured haemoglobin that protrude out of the erythrocyte membrane [62]. Subsequently, the kidney can be damaged by the Cu accumulation and the direct toxic effects of haemoglobin released following the red blood cell lysis, causing acute tubular injury [8,10,63]. Furthermore, degeneration of white matter in the brain and central nervous system gliosis can be developed [10,16,31,37,64,65]. Cu seems to cause *status spongiosus* in the sheep’s central nervous system as a result of altered glial transport mechanisms [64,65]. Chronic Cu intoxication is associated with death caused by both acute anaemia and haemoglobinuria-associated acute tubular injury [10]. It should be noted that the interruption in the ingestion of Cu does not avoid the development of a haemolytic crisis in the future. Therefore, surviving animals are not free of risk [10].

### 4.3. Acute Copper Poisoning

A Cu single dose of 20–210 mg/kg of body weight can cause acute toxicosis in sheep [10]. Oral acute exposure to Cu causes gastrointestinal irritation, necrosis and mucosal erosions [5,8]. The overage of this element and the resulting overload of Cu binding proteins, trigger an excess of free Cu ions in the cell that can damage nucleic acids and cellular proteins, and cause lipid peroxidation of membranes [8]. If the animal survives longer than 24–48 h, liver and kidney damage, and acute haemolytic crisis may develop [5,8,10].

When excessive Cu doses are injected, a rapid reaction is developed with high circulating Cu levels, and animals start to die. Early deaths are related to severe hepatic insufficiency because of centrilobular necrosis, while late deaths are due to renal failure caused by tubular necrosis [5,10].

## 5. Clinical Features

### 5.1. Chronic Copper Poisoning

CCP is the most common form of Cu toxicosis in sheep. A preclinical period, while Cu accumulates in the liver, precedes the acute onset of clinical signs characterizing this type of toxicosis, which does not constitute a chronic syndrome [5,10,16]. The following can be observed: pale mucous membranes, icterus, depression, weakness, recumbency, anorexia, excessive thirst, dyspnoea, arched back due to renal pain, as well as reddish brown to dark brown urine due to haemoglobinuria, and death after 24–48 h [5,8,10,16,30]. In addition, tachycardia, irregular pulse, tachypnoea, incoordination, ptyalism and nervous signs, such as blindness, tetraparesis, dementia and aggression, can be found in sheep suffering CCP [16,36,37,38,43,65]. Haywood et al. [66] described a CCP outbreak in which abortions, stillbirths and weakly lambs that often failed to thrive occurred in association with haemolytic crises.

### 5.2. Acute Copper Poisoning

In the case of acute Cu toxicosis associated with oral exposure to Cu, clinical signs consist of severe gastroenteritis, salivation, bluish-green diarrhoea containing mucus, and abdominal pain followed by dehydration, shock with low body temperature and tachycardia, and death [8,10,16]. If the animal survives longer and suffers a haemolytic crisis, it is accompanied by jaundice [8,10]. In the outbreak of acute intoxication as a result of ingestion of a Cu sulphate footbath described by Ortolani et al. [15], animals also showed anorexia, somnolence, depression and weakness, recumbency, the head turned to the flank, teeth grinding, moaning, tachypnoea, light-red urine, high thirst and slightly congestive mucus membranes.

If acute Cu poisoning is related to the parenteral administration of Cu preparations, anorexia, dehydration, depression, weakness and death are shown [5,10]. In addition, transient lip licking movements, soft faeces, diarrhoea, reduced appetite and tachypnoea have been seen in experimental studies [67]. In the case described by Mason et al. [47], some sheep showed central nervous signs, such as opisthotonos, head pressing or lack of awareness of surroundings, and dead sheep were found in groups along fences or in the scrub, indicating hepatic encephalopathy in association with liver injury.

## 6. Diagnosis

The diagnosis of Cu poisoning is based on history, clinical, gross pathological, histological and toxicological findings.

### 6.1. Chronic Copper Poisoning

#### 6.1.1. History and Clinical Findings

**Figure 1:**
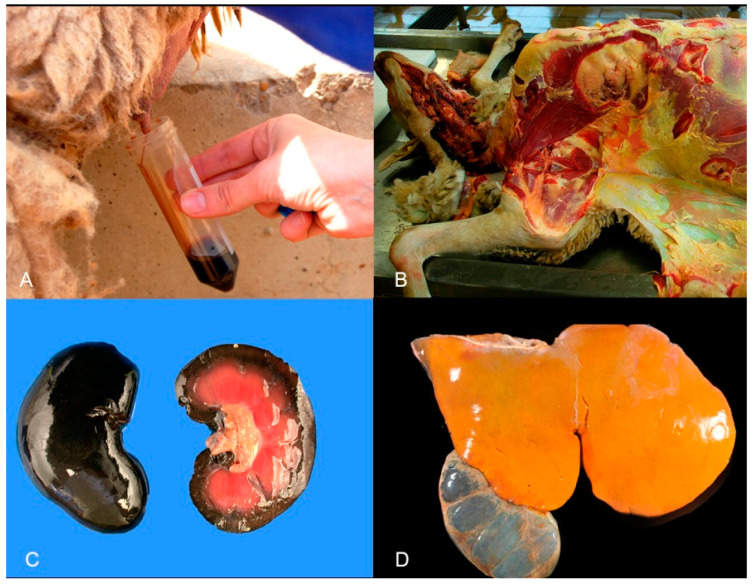
Sheep. Clinical signs and gross pathology of chronic copper poisoning. (**A**) Reddish brown urine. (**B**) The subcutaneous connective tissue showing an intense yellow colour interpreted as marked icterus. (**C**) Kidney. Dark “gunmetal” colour of the kidney surface and the renal cortex. Note the yellowish colour of the fat in the renal pelvis. (**D**) Liver. The organ shows a slight increase in size with rounded margins and orange-yellow discoloration. Note the dilated gallbladder filled with dark bile. Image 1D courtesy of Professor V. Pérez, León University, Spain.

The clinical disease follows a long subclinical period (weeks, months or even years) of Cu accumulation in the liver. It appears in a flock with the sudden onset of clinical signs associated with the development of an acute haemolytic crisis. As stated before, the main symptoms in affected animals are depression, weakness, anorexia, reddish brown to dark brown urine reflecting haemoglobinuria (Figure 1A), icterus, excessive thirst and teeth grinding, and a very high proportion of them die in 24–48 h [5,10]. As the sheep are prone to Cu poisoning, the disease must be kept in mind in sheep flocks with these clinical signs and other diseases such as babesiosis, leptospirosis, theileriosis, anaplasmosis, bacillary haemoglobinuria, *Clostridium perfringens* type A infection, poisoning by rape plant, kale, onion etc., that cause manifestations of haemolysis, must be considered in the differential diagnosis [10,36,39,62]. In the case of Cu toxicosis, the clinical disease often coincides with a period of stress on the animal (transportation, shearing, pregnancy, lactation, weather changes…), as any stressful condition predisposes to the release of Cu from hepatic stores [10,16,43]. One of the most important aspects to be investigated regarding the history is the possible excessive exposure to Cu for an extended period, and the more common sources of Cu have been described in the epidemiology section. But factors that alter Cu metabolism and can contribute to the development of CCP must also be taken into account: breed susceptibility to Cu [10,19,26,27]; the high efficiency of absorption in milk-fed lambs [12]; the bioavailability of Cu in the diet which is grater in processed feedstuffs [10,48,49]; a low intake of Cu antagonists, Mo, S, Zn and Fe [8,10,12,22,23]; or hepatic injury by toxic alkaloids from plants such as *Heliotropium europaeum* or *Senecia* sp., which affect Cu metabolism in the hepatocytes [12,16,21].

In the haemolytic phase, haematological findings typical of acute haemolytic anaemia are observed, with a sharp reduction in the packed cell volume (PCV), which can go down to values around 10% [43], red blood cell (RBC) count and haemoglobin, as well as macrocytosis, hypochromasia and basophilic stippling [16,30,37,42,43,65], which are common RBC abnormalities in regenerative anaemia of small ruminants [62]. Heinz bodies and methaemoglobinemia have also been referred [16,35,37,43,68], which can appear in haemolytic anaemias from oxidant injury to RBCs, in this case caused by the sudden increase in blood Cu concentrations [22,62].

Biochemical analysis reveals significant increases in plasma bilirubin due to the haemolytic crisis, and also in liver-specific enzymes as a consequence of Cu hepatotoxicity [10,16,30,35,37,39,65]. A marked increase in serum hepatic enzymes from the damaged liver, glutamate dehydrogenase (GLDH), aspartate aminotransferase (AST), gamma-glutamyltransferase (GGT), sorbitol dehydrogenase (SDH) and alkaline phosphatase (ALP), occurs immediately prior the onset of the haemolytic crisis [10,13,30,49,69]. But significant increases have also been observed throughout the Cu loading phase, and the assessment of these enzymes has been used for the early detection of CCP. The first enzyme to be proposed was AST [70]. Subsequent studies demonstrated that serum levels of AST and GGT increased very early during the Cu loading, 9 weeks before clinical signs, while ALP level was unreliable for early diagnosis [30]. Other authors have evaluated the levels of GLDH, GGT and AST in sheep exposed to a high oral Cu intake and found a high correlation between GLDH activity in plasma and liver Cu concentrations, concluding that GLDH had the highest sensitivity of the three enzymes for the diagnosis of CCP [71]. In a further study, Ortolani et al. [13] measured AST, GGT and SDH levels in experimentally induced Cu poisoning in sheep. They found that GGT and AST levels started to increase 28 and 14 days, respectively, before the haemolytic crisis, and these increases continued steadily in the prehaemolytic period, whereas fluctuating SDH levels were measured. These authors concluded that GGT followed by AST are the best enzymes to assess Cu load in sheep during the prehaemolytic period, and could be used for early diagnosis and evaluation of the prognosis of Cu poisoning [13]. More recently, increased serum GGT and AST activity in sheep receiving a high Cu supply was measured from the eleventh week, and was demonstrated to be influenced by Cu supplementation [51]. However, although AST and GGT can be used for early diagnosis of Cu poisoning, other studies have concluded that these enzymes could not be used for the prediction of mortality [39,72]. Notwithstanding the utility of these enzymatic tests, it should always be noted that they are not specific to Cu toxicity, and liver damage can be due to other conditions such as the presence of parasites.

Cu poisoning can lead to kidney damage caused by free Cu and haemoglobin [8,10,63], and increased serum levels of urea and creatinine, and also proteinuria can appear following the haemolytic crisis [30,37,42,43]. Serum total protein can also decrease when liver and kidney damage is severe [37].

#### 6.1.2. Necropsy and Histopathological Findings

The most characteristic necropsy findings in CCP are manifestations of the haemolytic crisis, including icteric tissues (Figure 1B), swollen dark-coloured kidneys (Figure 1C), reddish brown to dark brown urine in the urinary bladder and enlarged and soft spleen with brown-black parenchyma. The liver is usually large, friable and yellowish, and the gallbladder is distended, containing dense and dark bile (Figure 1D). Variable amounts of fluid in body cavities are also reported [5,10,16,17,31,36,37,38,43].

**Figure 2:**
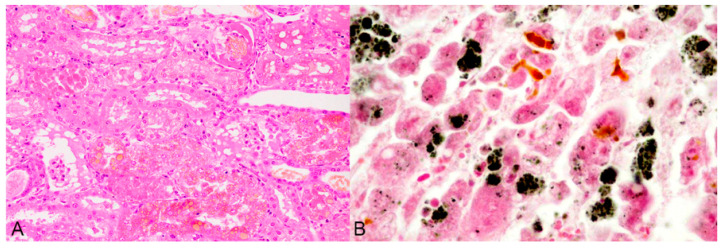
Sheep. Histopathology of chronic copper poisoning. (**A**) Kidney. The renal cortical tubular epithelial cells with vacuolated appearance, degeneration and necrosis. Note the orange-red hyaline material, interpreted as a haem compound, within the vacuoles of some cells. The same material is also found free within the lumen of renal tubuli. Haematoxylin and eosin. Obj. 40×. (**B**) Liver. Vacuolar degeneration and necrosis of the hepatocytes. Black round spots of different sizes, indicating copper granules, are found dispersed or condensed into the cytoplasm of hepatic cells. Note the dark yellow pigment within the bile canaliculi. Rubeanic acid stain. Obj. 40×. Image 2B courtesy of Professor V. Pérez, León University, Spain.

As a consequence of haemolytic crisis, there is considerable kidney damage, and histopathological findings are typical of haemoglobinuria-associated acute tubular injury (formerly known as haemoglobinuric nephrosis), with proximal tubular epithelial cells with vacuolated appearance, degeneration and necrosis, and tubular lumens, mainly those of the proximal tubules, filled by abundant orange-red granular refractile material, the characteristic appearance of a haem compound (Figure 2A). Haemoglobin can be confirmed by Okajima stain. Glomerular basal lamina and capillary blood vessels could also be damaged [16,17,30,31,37,38,43,63,73].

The liver histological changes during the Cu loading phase reflect a chronic hepatic injury, and include hepatic parenchymal swelling, hepatocytes with vacuolated appearance, necrosis of isolated parenchymal cells, proliferation of epithelial cells of bile ducts and more numerous Kupffer cells. Haemolytic crisis causes extensive hepatocellular necrosis affecting centrilobular and midzonal regions, but massive necrosis can occur in severe cases [10,16,19,23,30,31,37,38,43,74,75]. Excess Cu in the liver can be confirmed by rubeanic acid or rhodanine stain (Figure 2B). These staining techniques detect Cu granules in the cytoplasm of the hepatocytes as the cellular mechanism for Cu storage becomes overwhelmed. A significant linear relationship has recently been demonstrated between increasing hepatic Cu concentrations and the abundance of rhodanine-stained granules observed in histological sections from liver tissue [76], and the potential of this technique as an indicator of excess Cu concentrations has been highlighted [9].

Some authors also describe histological changes in the spleen, such as hemosiderosis and congestion [16,31,37,43], and the brain, such as degenerative lesions in the cerebellum and cerebrum [10,16,31,37,64,65].

#### 6.1.3. Toxicological Analysis

The tentative diagnosis of CCP obtained with history, clinical, gross pathological and histological findings is definitively confirmed by the elevated concentration of Cu in tissue samples, mainly liver, kidney, blood and serum or plasma. Cu determination could be performed, after initial acid digestion (not necessary for serum or plasma), by the atomic absorption or flame atomic absorption spectrometric method [77], or by inductively coupled plasma optical emission or inductively coupled plasma mass spectrometry [78]. Cu can also be determined in serum or plasma spectrophotometrically using a clinical chemistry analyser [38].

Cu determination in blood, plasma or serum is not a reliable diagnostic tool for CCP. Little correlation exists between hepatic Cu concentrations and Cu concentration in blood [1]. In the haemolytic phase, blood Cu rises significantly above normal due to the releasing of Cu from the liver [8,10,13,18,49], but this increase is transient and declines quickly if the animal survives, mainly following fluid therapy [8]. During the prehaemolytic phase, blood Cu concentration remains unchanged or even decreases, and starts to elevate only 24 h before the haemolytic crisis, so it is not a useful parameter for diagnosing Cu loading phase [13,49,69,71]. Post-mortem testing for Cu in the liver and kidney is the best diagnostic indicator in the clinical phase [8,10,17,42,72], and should be performed in both tissues for a reliable diagnosis of Cu poisoning, as mobilization of Cu from the liver may reduce concentrations to normal levels but the mobilised Cu is then accumulated in the kidney [8,10,17]. Cu toxicosis may be associated with hepatic and renal concentrations of Cu greater than 500 and 100 mg/kg DM, or 150 and 15 mg/kg on a wet matter basis (WM), respectively [10,17,45]. Cu concentrations in liver and kidney samples at the time of haemolytic crises reported in the literature are very variable, although in most cases liver Cu concentrations are higher than 1000 mg/kg DM [13,16,17,35,36,37,38,43,49,65,74]. However, histological evidence of liver damage has been reported at liver Cu concentrations as low as 350 mg/kg DM [79]. On the other hand, elevated liver concentrations confirm excessive exposure to Cu but do not necessarily indicate clinical Cu toxicity. Because of that, a marginally toxic range of 400–1000 mg/kg DM (135–338 mg/kg WM) in the liver and 38–50 mg/kg DM (13–17 mg/kg WM) in the kidney has been proposed [12]. Values below the lower limit rule out CCP, values above the upper limit strongly suggest CCP and marginal values indicate probable CCP [12]. Some authors have suggested that for a reliable diagnosis, Cu determinations should be performed on a dry matter basis, as the state of dehydration can affect the Cu DM/Cu WM ratio [17]. Cu determination in liver biopsy samples from live animals, obtained by aspiration biopsy or surgical techniques (paracostal laparotomy), allows the assessment of hepatic Cu accumulation during the prehaemolytic phase [7,33,51,71,80].

Toxicological analysis of pastures, feedstuffs, mineral supplements, water or the close environment of the sheep for sources of Cu can also contribute to the diagnosis.

### 6.2. Acute Copper Poisoning

The disease is much less frequent in sheep than the cumulative form and may arise soon after a single or small number of large quantities of Cu complexes are ingested orally or administered parenterally. As already indicated, it is usually seen after accidental administration of excessive amounts of Cu salts present in anthelmintics and in preparations for the prevention and treatment of Cu deficiency, but there are also descriptions related to the ingestion of mineral mixes formulated for other species, improperly formulated rations, or Cu sulphate footbaths [5,10,11,15,16,45].

Acute Cu poisoning due to ingestion is mostly related to severe gastroenteritis. Faeces often contain large amounts of mucus and have a characteristic blue-green colour, which helps to make a preliminary diagnosis of the process, discarding other causes of gastroenteritis. Severe shock with a fall in rectal temperature, tachycardia, depression and weakness appear, and animals can die in a few hours [5,10,15]. If acute toxicosis arises by Cu injection, the response is more rapid and digestive signs are not present, the most important symptoms being severe depression and dehydration [5,10,46,47,67]. In both forms, if the animal survives sufficiently long, haemoglobinuria and jaundice can be observed as a consequence of haemolysis [5,10,15]. However, in a case of acute toxicosis by ingestion of Cu sulphate footbath, a lower degree of haemolysis, as compared to sheep with CCP, has been reported, with a higher packed cell volume and consequently less intense haemoglobinuria [15].

Markedly elevated circulating levels of bilirubin and liver enzymes AST and GGT have also been measured, consistent with liver damage [15,47]. In this sense, some authors speculate that the origin of elevated bilirubin levels in acute toxicosis is hepatic rather than prehepatic, unlike what happens in the haemolytic crises of chronic poisoning, and higher AST activity than in sheep with cumulative Cu poisoning has also been reported [15]. Elevated levels of blood urea [15,67] and creatinine [15] could be present, reflecting the deterioration of renal function.

Necropsy findings reflect the route of intoxication and rapidity of onset, and include the presence of fluid in body cavities, swollen liver and kidneys and, in cases of Cu ingestion, lesions in the gastrointestinal mucosa such as erosions, ulcers, petechial and ecchymotic haemorrhages, mainly in the abomasum and duodenum [5,10,15]. Gastrointestinal lesions appear as a consequence of the corrosive effect of divalent Cu, ionised by the low abomasum pH [15]. The characteristic histological finding in the liver is severe and generalised hepatocellular necrosis [10,15,47,67], whereas histological descriptions of kidneys reveal renal injury of variable intensity [10,15,67].

Apart from these findings, diagnosis is confirmed by the raised concentrations of Cu in serum liver and kidney, although this increase occurs days after oral poisoning, and in this case, large amounts of Cu can also be detected in the stool [10]. Kidney Cu should be determined after a massive dose because the kidney can reach elevated Cu concentration before the liver [10]. In acute poisoning by Cu ingestion, some authors have reported serum Cu levels much lower than in the acute haemolytic crisis of cumulative form, which would explain the lower rate of haemolysis, as well as lower liver Cu concentration, despite the fact that significant liver injury was observed, probably caused by the free ionised Cu absorbed suddenly in large amounts [15]. There are even descriptions of liver Cu levels within the normal range in sheep dying following the injection of a single small dose of Cu compounds that caused hepatotoxic effects [47,67].

## 7. Treatment and Prevention

### 7.1. Chronic Copper Poisoning

Different therapies have been used to treat and prevent CCP in sheep based on either chelating agents or Cu antagonists.

Treatment of sheep with severe clinical signs following a haemolytic crisis has had a poor success rate [8,11], although treatments with the Cu antagonists Mo and S, or directly with tetrathiomolybdate, the mediator of the Cu–Mo–S antagonism, have been demonstrated to be effective in preventing death in some animals already in the haemolytic phase. These therapies must be accompanied by supportive care, including fluid therapy, and a blood transfusion should also be considered. Ammonium or sodium molybdate (50–500 mg) and sodium thiosulphate (0.3–1 g) should be used daily for up 3–4 weeks as a drench or administrated orally [8,14,17]. Ammonium tetrathiomolybdate (ATTM) has shown the greatest effectiveness in reducing mortality rates in animals with clinical signs of Cu toxicosis. It has been regarded as the treatment of choice, promoting the rapid clearance of the chelated complex (Cu-ATTM) by the biliary–faecal route and reducing hepatic Cu levels [6,81,82]. It can be administered intravenously, at 2.7 mg/kg for 3–6 treatments separated by 2–3 days [10,81,83], but the most convenient treatment involves subcutaneous injections of 3.4 mg/kg of ATTM on three alternate days [1,8,11,84].

However, when clinical signs of CCP are identified in individual sheep from a group, it is likely that other animals will have subclinical liver damage. Treating subclinical cases in the prehaemolytic period is crucial for the control of the poisoning outbreak, as treatment is very effective in preventing haemolytic crisis in at-risk sheep [11,17,35,72,81,84]. Different treatment regimens for use in field outbreaks of sheep CCP have been proposed based on molybdate compounds. ATTM treatment on a flock basis, administered intravenously [83] or by subcutaneous injection, as described above, and more convenient in terms of cost and applicability [84], has shown to be very effective in preventing further clinical cases and has been widely used [10,11,66,83,84]. However, the product is difficult to obtain, and some countries’ laws, such as those that belong to the European Union, prohibit its use in food-producing animals [11]. Long-term pathological consequences of the treatment with ATTM of Cu-poisoned sheep have also been reported, associated with a toxic pituitary endocrinopathy, in which the animals became infertile and progressively unthrifty, and died 2–3 years later [66,85].

Oral therapies with ammonium molybdate and sodium thiosulphate, or ammonium or sodium molybdate alone, have also been recommended for the treatment of all at-risk sheep in flocks with cases showing haemolytic signs, obtaining beneficial results [16,17,30,35,43,72,86]. These treatments are cheap, widely available and can be administered via concentrates. The treatment should be offered to all susceptible sheep, although some authors recommend treating all animals, including those that are sick [17]. Other approaches consider it essential to identify and determine the percentage of animals in the flock that are prehaemolytic and those that are already haemolytic because this determines the treatment that must be applied, especially in large flocks [35]. The assessment of hepatic enzymes (AST, GGT) is a non-specific test that enables the identification and timely treatment of preclinical cases, as increased activities of these enzymes are present several weeks before a haemolytic crisis [13,30]. The subsequent decrease in these liver enzymes in treated animals is a useful indicator of the effectiveness of the treatment applied, together with the cessation of the clinical cases [35,39,72]. In addition to treating subclinically affected animals, exposure to the Cu source should be eliminated, and animals should be transferred to a diet with lower available Cu content [1,12].

Therapies using other chelating agents such as D-penicillamine (PEN), sodium calcium edetate or 2-3 dimercapto-1-propanol have also been experimentally tested to treat Cu-loaded sheep, with variable success. Different studies shown the positive effect of PEN, a drug used in children with Wilson’s disease, a genetic disorder of Cu metabolism [87], on the Cu excretion in affected sheep when administered orally or intraperitoneally, and it has been proposed for the treatment of Cu toxicosis in sheep [72,82,88,89]. Unfortunately, the cost of PEN may be prohibitive for the general treatment of a flock [72,82,88]. Apart from this, PEN is not specific to Cu and also removes Zn [82]. A possible treatment with sodium calcium edetate, commonly used for the treatment of lead poisoning in ruminants, injected subcutaneously or intravenously (60–75 mg/kg), has also been suggested [10,11], although some studies have shown that the treatment in experimental animals caused no significant effect on the Cu excretion [82,88]. On the other hand, the traditional chelating agent 2-3 dimercapto-1-propanol, or its derivative 2-3 dimercapto-1-propane-1-sulphonate, also did not seem to have a Cu mobilizing effect to any significant degree [82,88].

A prophylactic measure to prevent Cu hepatic accumulation and reduce the risk of CCP in vulnerable breeds, in areas where the disease is endemic, or in intensively managed lambs or milk sheep, is the addition of Cu antagonist supplements to the ration to lower the availability of dietary Cu [1,5,11,12]. In the reducing environment of the rumen, dietary Mo and S naturally form thiomolybdate compounds which interact with available Cu in the digestive tract forming an insoluble precipitate and greatly reducing Cu availability [8,9,55,90]. There is also a Fe–Cu–S interaction in the rumen, and it does decrease Cu availability [55]. Zn effect is related to the induction of MT in the intestine, limiting intestinal Cu absorption [7].

It has been demonstrated that dietary supplementation with Mo and S reduces hepatic Cu accumulation in sheep [91,92]. Fe supplementation also reduced liver Cu in cattle [93]. No effect was noted in sheep, but in this experiment, animals received a low-S diet [94]. The usefulness of Zn for protecting sheep against CCP has also been shown [95], reducing the hepatic Cu load [7,53]. However, Zn supplementation, maintaining the Zn–Cu ratio (6:1), did not seem to be sufficiently effective in preventing liver damage caused by increased dietary Cu concentrations [32]. In order to reduce the risk of toxicity, it is common the use mixtures of antagonists [1,12,80]. The reduction of the maximum permitted levels (mpl) in the European Community has also contributed to this trend, although there is little information about the additivity of protective effects. In this sense, a study demonstrated that a blend of Mo, S and Zn at mpl reduced liver Cu accretion in lambs of a vulnerable sheep breed, but insufficiently to avoid mild hepato-toxicity, which was resolved upon feeding a Cu depletion regimen [80].

In addition to the oral route, other routes of administration of Cu antagonists with prophylactic purposes have been evaluated. A commercial molybdate formulation (containing 42 mg Mo) injected subcutaneously two times 42 days apart did not prevent hepatic Cu accumulation in sheep receiving Cu supplementation for 10 weeks [96]. Another treatment with two Zn oxide boluses (43 gr Zn oxide) administered via a rumen cannula 42 days apart also failed to do so [96]. Another study developed in Norway, where sheep Cu poisoning is an endemic disease encountered under normal grazing conditions, confirmed the effect on the short-term Cu hepatic stores of the widely used treatment with three subcutaneous injections of ATTM. But this treatment had a little prophylactic effect on the long-term Cu liver accumulation [33].

These therapies to prevent Cu hepatic accumulation need subsequent monitoring to avoid the risk of inducing a Cu deficiency state. Some authors have indicated that their use should be discontinued as soon as sensitive indicators of liver damage, such as hepatic enzymes, indicate that hepatotoxicity has been eliminated [80].

It has also been proposed that appropriate genetic selection could lower the susceptibility of vulnerable breeds to CCP. Selection of Texel sires for low plasma Cu resulted in lower liver Cu in their offspring, and a significant effect was also found by selecting sires for low liver Cu status [1,12,77].

Periodic liver sampling, from cull animals or from biopsy, as a measure of flock Cu status, can help in the making of more effective long-term decisions in order to reduce the risk of CCP [9].

### 7.2. Acute Copper Poisoning

The treatment of animals acutely poisoned with Cu mainly consists of supportive treatments directed at the shock, dehydration and damage to the gastrointestinal tract [8,10]. The gradual recovery of seriously ill sheep when treated, in addition to this, with ATTM (3.4 mg/kg) intravenously administered and a drench containing 150 mg ammonium molybdate and 1.5 g sodium sulphate has also been reported. This treatment was given once daily for four days [15].

Acute Cu poisoning is mainly prevented by ensuring an accurate dosage of Cu preparations [11].

## 8. Conclusions

Sheep are particularly susceptible to Cu intoxication, a severe disease widespread in sheep flocks worldwide, causing significant economic losses due to the high mortality rates. The incidence of CCP, the most common form of the disease caused by chronic exposure to Cu, is increasingly linked to the use of more intensive methods of sheep production. Treating animals with severe clinical signs often has a poor success rate, and identifying and treating subclinical cases is crucial for controlling Cu poisoning outbreaks in sheep flocks. The adoption of preventive measures to reduce the risk of CCP, such as dietary supplementation with Cu antagonists, could be required in vulnerable breeds or in intensively managed lambs or milk sheep. The measure of flock Cu status by periodic liver sampling is a valuable tool for decision making.

## Data Availability

Not applicable.
